# Label-Free Digital Holo-tomographic Microscopy Reveals Virus-Induced Cytopathic Effects in Live Cells

**DOI:** 10.1128/mSphereDirect.00599-18

**Published:** 2018-11-21

**Authors:** Artur Yakimovich, Robert Witte, Vardan Andriasyan, Fanny Georgi, Urs F. Greber

**Affiliations:** a Department of Molecular Life Sciences, University of Zurich, Zurich, Switzerland; b MRC Laboratory for Molecular Cell Biology, University College London, London, United Kingdom; University of Michigan—Ann Arbor; Institut Pasteur; Pennsylvania State University

**Keywords:** apoptosis, cell contraction, cell volume, herpes simplex virus, label-free microscopy, live-cell microscopy, membrane blebbing, refractive index, rhinovirus, tomography, vaccinia virus, virus infection

## Abstract

Cytopathic effects (CPEs) are a hallmark of infections. CPEs are difficult to observe due to phototoxicity from classical light microscopy. We report distinct patterns of virus infections in live cells using digital holo-tomographic microscopy (DHTM). DHTM is label-free and records the phase shift of low-energy light passing through the specimen on a transparent surface with minimal perturbation. DHTM measures the refractive index (RI) and computes the refractive index gradient (RIG), unveiling optical heterogeneity in cells. We find that vaccinia virus (VACV), herpes simplex virus (HSV), and rhinovirus (RV) infections progressively and distinctly increased RIG. VACV infection, but not HSV and RV infections, induced oscillations of cell volume, while all three viruses altered cytoplasmic membrane dynamics and induced apoptotic features akin to those caused by the chemical compound staurosporine. In sum, we introduce DHTM for quantitative label-free microscopy in infection research and uncover virus type-specific changes and CPE in living cells with minimal interference.

**IMPORTANCE** This study introduces label-free digital holo-tomographic microscopy (DHTM) and refractive index gradient (RIG) measurements of live, virus-infected cells. We use DHTM to describe virus type-specific cytopathic effects, including cyclic volume changes of vaccinia virus infections, and cytoplasmic condensations in herpesvirus and rhinovirus infections, distinct from apoptotic cells. This work shows for the first time that DHTM is suitable to observe virus-infected cells and distinguishes virus type-specific signatures under noninvasive conditions. It provides a basis for future studies, where correlative fluorescence microscopy of cell and virus structures annotate distinct RIG values derived from DHTM.

## Abstract (precis)

This study introduces label-free digital holo-tomographic microscopy (DHTM) and refractive index gradient (RIG) measurements of live, virus-infected cells. We use DHTM to describe virus type-specific cytopathic effects, including cyclic volume changes of vaccinia virus infections, and cytoplasmic condensations in herpesvirus and rhinovirus infections, distinct from apoptotic cells. This work shows for the first time that DHTM is suitable to observe virus-infected cells and distinguishes virus type-specific signatures under noninvasive conditions. It provides a basis for future studies, where correlative fluorescence microscopy of cell and virus structures annotate distinct RIG values derived from DHTM.

## INTRODUCTION

Viruses have a dual nature, the particle and the infected cell. At the onset of an infection, the particle introduces proteins, DNA or RNA, and sometimes lipids into the host cell. The infected cell either produces viral components that are encoded in the viral genome or raises an immune reaction against the virus and silences the infection. In the former case, the infected cell develops a cytopathic effect (CPE). CPEs are diagnostic hallmarks of a particular virus, and it is well-known that CPEs occur in cell cultures (for reviews, see references 1 and 2). CPE can predict clinical outcomes *in vivo*. Examples include exacerbation of steatosis by hepatitis C virus, apoptosis in trigeminal ganglia by herpes simplex virus (HSV), or aseptic meningitis, paralysis, cardiomyelitis, and herpangina by enteroviruses, including poliovirus, coxsackievirus, and enterovirus (EV) type 71 (3–5). While virus-induced CPE and cell death are exacerbated by cytokine responses, cytotoxic T cells, or natural killer (NK) cells, virus-induced CPE of cultured cells apparently proceeds in a cell-autonomous manner.

The nature and the extent of CPE depend on the virus, cell type, host innate response, and progression of infection. For example, distinct levels of CPE correlate with the amounts of newly synthesized virus particles (6). In adenovirus-infected cells, the cells may lyse and release large amounts of viral particles after the cells show strong CPEs, while persistently infected cells produce small amounts of progeny over time (long term) and have weak CPEs (7, 8). Yet, the extent of CPE does not always correlate with virion production, as cells undergoing programmed cell death feature strong CPE at low viral titers (9–11). As viruses hijack cellular resources, CPEs elicited by virus infection may have distinct features, such as loss of membrane integrity, cell shrinkage, increased chromatin density, cell detachment from the substratum, formation of syncytia, loss or enforcement of the cytoskeleton, and reorganization of intracellular membranes (2, 12, 13). Despite the predictive nature of CPE for clinical and biological infections, time-resolved 3D analyses of virus-induced CPE are missing.

Here we describe a new approach using 3D digital holo-tomographic microscopy (DHTM) to study the CPEs induced by three different viruses: vaccina virus (VACV), a large DNA virus replicating in the cytoplasm, herpes simplex virus type 1 (HSV-1), a large DNA virus replicating in the nucleus, and rhinovirus (RV), a small RNA virus replicating on cytoplasmic membranes. Classical video-enhanced contrast optical microscopy, such as interference microscopy and bright-field microscopy, are limited by uneven image field intensity, lack of tomographic information, and a focus-dependent size inflation of structures due to diffraction limitation (14). In contrast, DHTM allows prolonged quantitative time-resolved 3D image acquisition using an ultralow-powered laser (520-nm class 1 with 0.2 mW/mm^2^) without recognizable phototoxicity at high temporal and spatial resolution (15).

## RESULTS

**FIG 1:**
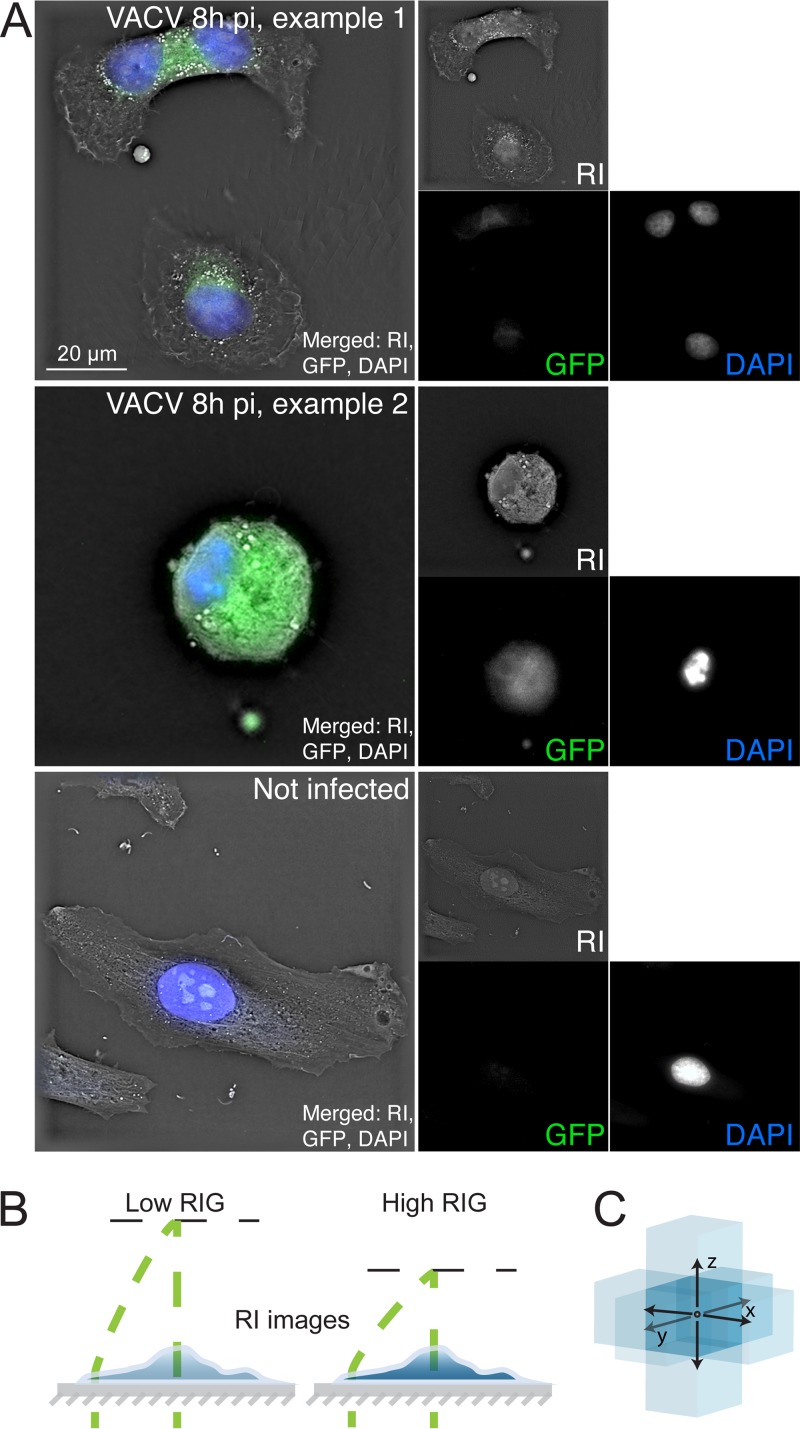
Correlative digital holo-tomographic microscopy and light microscopy of fixed and permeabilized cells. (A) Representative images of HeLa-ATCC cells incubated with 2 ml RPMI at 37°C for 8 h, fixed in 4% PFA in PBS, and imaged with a DHTM microscope and an attached epifluorescence module. The cells were either infected with VACV expressing GFP under the control of early/late promoters (VACV_E/L-GFP; top and middle panels) at an MOI of 2 or left uninfected (bottom panel). The top panels depict a VACV-GFP-infected cell in early infection stage. The middle panels depict a rounding cell, indicating late**-**stage VACV infection. The bottom panel depicts an uninfected cell. RI is displayed in gray, GFP is shown in green, and nuclei stained with DAPI appear in blue. Bar, 20 µm. (B) Schematic illustrations of RI computation. A cell can be thought of as a gradient index microlens, changing its optical properties depending on biochemical activities. (C) RIG is derived from RI and represents a voxel-based measurement of the difference of the refractive index in 3D space. The RIG value of the voxel in the middle is represented as a middle blue box, which is calculated based on the difference to the light blue voxels in the 3D neighborhood. Note that the reference beam (curved green dashed line) does not pass through the sample. RI is based on changes between the beam (straight green dashed line) and the reference beam.

We infected HeLa cells with the VACV strain Western Reserve harboring an early/late GFP expressed transgene (VACV_WR E/L-GFP [VACV-GFP]) at a multiplicity of infection (MOI) of 2, yielding >95% infected cells, fixed the cells 8 h postinfection (pi) with paraformaldehyde, and recorded the refractive index (RI), nuclear DAPI stain, and GFP intensity by correlative DHTM and fluorescence microscopy and compared the infected cells to noninfected cells. At 8 h pi, all cells inoculated with VACV-GFP were infected based on their GFP intensity and DAPI staining of cell nuclei, whereas the GFP intensity of the uninfected cells was in the range of the background (Fig. 1A). Based on the RI change across a volume, the RI gradient (RIG) can be computed across the whole cell, similar to the RIG across an index gradient lens (for a simplistic illustration, see Fig. 1B and C).

**FIG 2:**
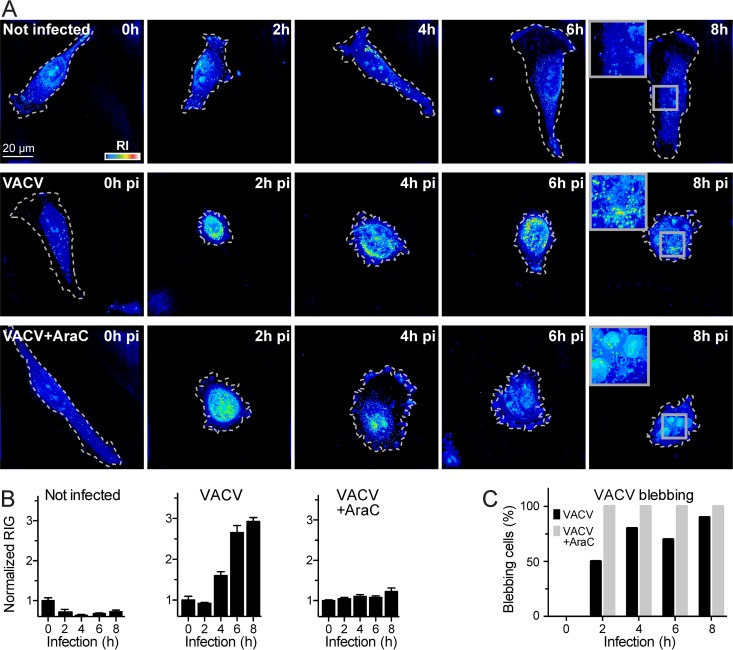
VACV late gene expression increases the cellular RIG. (A) Representative images of HeLa-ATCC cells incubated with 2 ml RPMI at 37°C for 8 h and imaged at 2**-**h intervals by DHTM. Cells were uninfected, infected with VACV-GFP at an MOI of 2, or infected with VACV-GFP at an MOI of 2 in the presence of 10 µM AraC. The perimeters of the cells grown on coverslips are outlined with white dashed lines. RIs are depicted as intensity values in a “thermal” lookup table. Images were obtained as holograms and depicted as projections of maxima along the *z* axis of the 3D stacks. Bar, 20 µm. (B) Cell RIG quantification of panel A. RIG values were normalized to 1 for 0 h postinfection (pi). Bars depict mean values plus SEM (error bars) of at least 10 cells for each condition and time point. (C) Comparison of the frequency of the “blebbing” phenotype in VACV-GFP-infected cells and VACV-GFP-infected cells in RPMI containing 10 µM AraC. Data from at least 10 cells per time point and condition were acquired and manually scored as blebbing or nonblebbing.

We next imaged VACV-GFP-infected and uninfected cells in live-cell mode by DHTM in 2-h intervals for up to 8 h pi (Fig. 2A). For each condition, two dishes were infected as described in Materials and Methods. At least five randomly chosen cells from each dish were imaged, yielding at least 10 cells per time point and condition. A progressive and prominent change in RI was observed in the infected cells, visualized in scaled pseudocolor. In contrast, the uninfected cells and the VACV-GFP-infected cells treated with the deoxynucleoside analogue cytarabine (AraC), an inhibitor of VACV late gene expression (16, 17), showed less prominent RI changes, although AraC-treated cells inoculated with VACV-GFP exhibited a strong increase in RI in the cell nucleus. We quantified the RI change by deriving the RIG values across the entire cell (Fig. 2B), as described in Materials and Methods. The RIG values of the VACV-GFP-infected cells gradually increased as the infection progressed, reaching threefold at 8 h pi compared to the RIG prior to infection. In contrast, the RIG values of uninfected cells remained largely constant over 8 h. The AraC-treated cells showed only a small RIG increase of about 1.3-fold, suggesting that the RIG increase is predominantly due to viral late gene expression. VACV infections exhibit membrane blebbing phenotypes at early and late time points, as well as focal bud-like swellings (18). Such features are diagnostic of a contractile cell cortex, as reported for uninfected cells in cell migration and response to mechanical cues (19). We noticed an increased fraction of blebbing cells over 2 to 8 h pi in the presence of AraC (Fig. 2C). This is distinct from membrane blebbing during entry (20) and suggests that blebbing at 8 h pi does not require viral late gene expression and does not affect the RIG.

**FIG 3:**
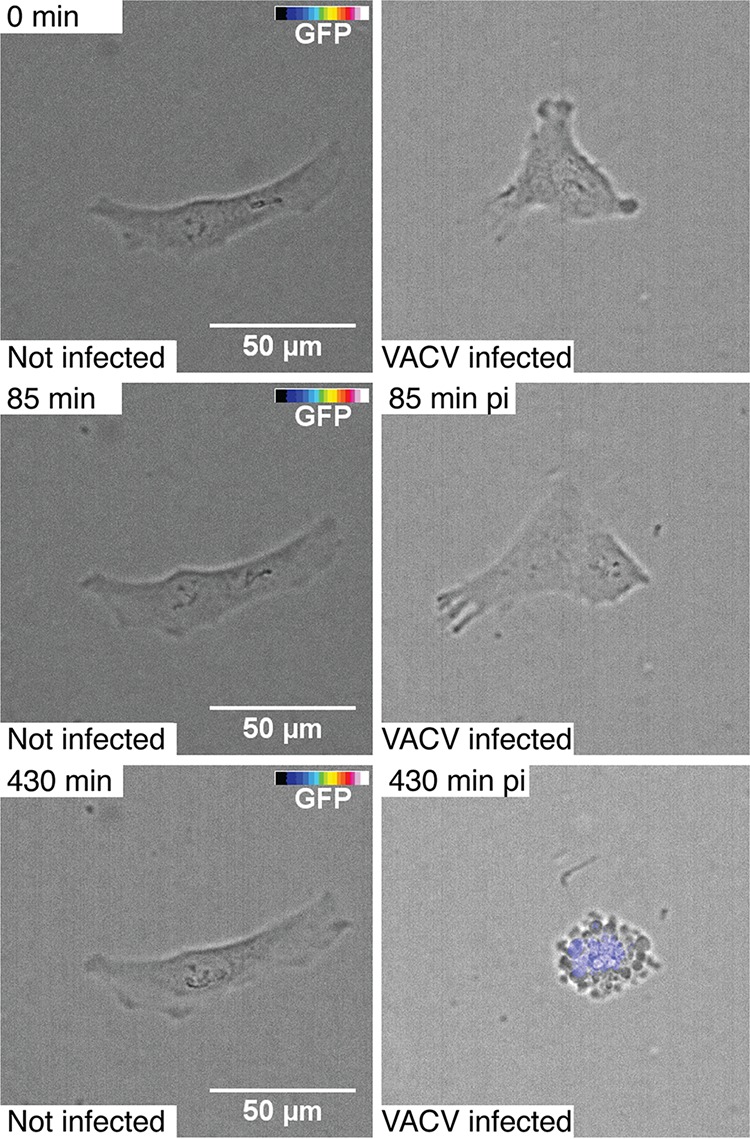
Cell morphology and VACV-GFP transgene expression visualized by automated and correlative phase-contrast and fluorescence live-cell time-lapse microscopy. HeLa-ATCC cells were uninfected (left panels) or infected (right panels) with VACV-GFP using the cold binding protocol (30-min inoculation on ice, wash, and transfer to 37°C). Cells were imaged with a high-throughput wide-field microscope every 5 min for 8 h. Cells were visualized with transmission light, the GFP fluorescence was intensity color coded (color bar ranging from transparent through blue to white). Bars, 50 µm. See also Video S1 in the supplemental material.

To test whether similar morphology changes of the VACV-GFP-infected cells can be observed with an alternative imaging modality, we infected HeLa cells with VACV-GFP at an MOI of 2 and imaged live cells in phase-contrast and fluorescence modes at 5-min intervals for 8 h (Fig. 3; see also Video S1 in the supplemental material) by using a high-throughput light microscope. In agreement with the DHTM imaging results, the VACV-GFP-infected cells expressed the GFP transgene at increasing intensity over the course of infection. As recorded in the DHTM experiments, we observed host cell rounding, contractions and late stage blebbing of the infected cells, while the features of the uninfected cells remained largely invariant.

10.1128/mSphereDirect.00599-18.2 VIDEO S1 Cell morphology and VACV E/L-GFP transgene expression visualized by automated and correlative phase-contrast and fluorescence live-cell time-lapse microscopy. HeLa-ATCC cells were either mock infected (left panel) or infected (right panel) with VACV_E/L-GFP virus (MOI of 2). Cells were imaged with a high-throughput wide-field microscope every 5 min for 8 h. Samples were visualized with transmission light. The GFP fluorescence was color coded (color bar corresponding to fluorescence intensity from transparent through blue to white). Scale bar indicates 50 µm. The video is related to Fig. 3. Download Video S1, MOV file, 5.8 MB. Copyright © 2018 Yakimovich et al. 2018 Yakimovich et al. This content is distributed under the terms of the Creative Commons Attribution 4.0 International license.

**FIG 4:**
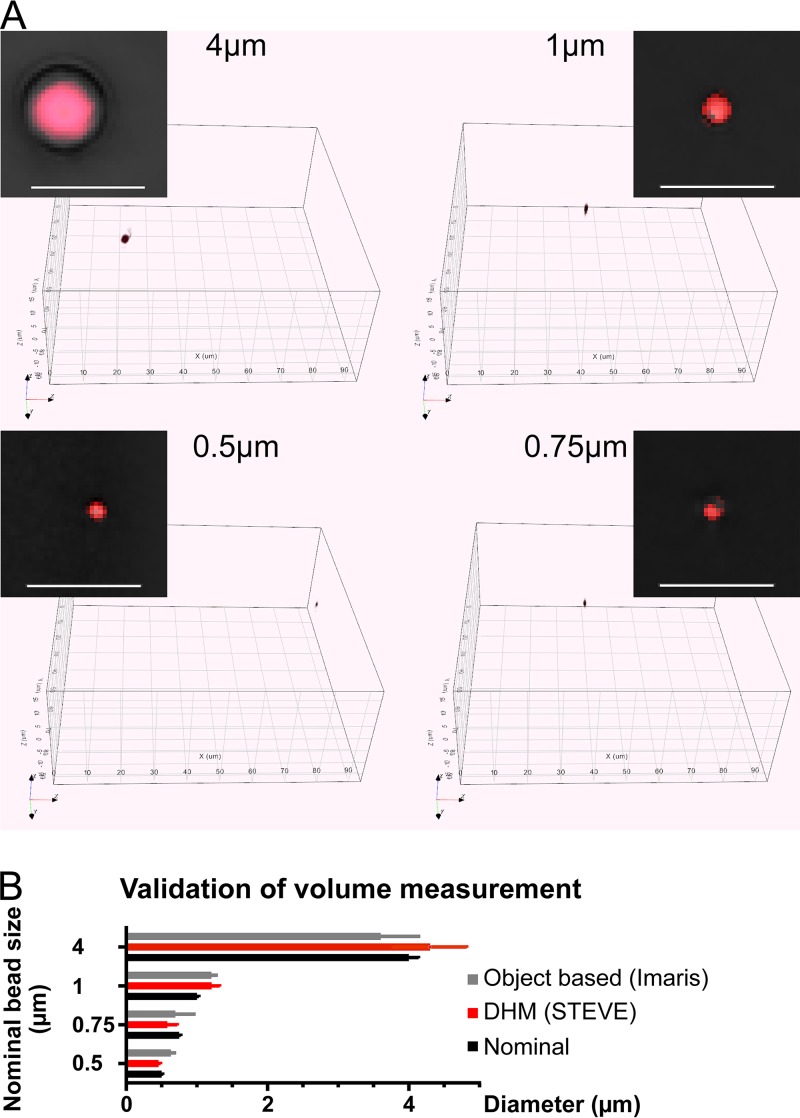
Benchmarking the volume measurement using polystyrene beads of defined size. (A) Polystyrene beads (Tetraspeck; ThermoFisher) of defined diameter were diluted in PBS, allowed to sediment to the bottom of the dishes, and imaged using a 3D Cell Explorer microscope. The small colored boxes depict digital RI stain of beads in red on black background. The large boxes show full 3D visualization of acquired holographs. 3D stacks of the beads were digitally stained (voxel segmented) for an RI estimated to cover at least 95% of the bead volumes. Bars, 5 µm. (B) Comparison of volume quantification performed by voxel summation either by voxel segmentation and counting in STEVE software or by surface fitting in Imaris. Bead diameter was computed based on voxel counts and voxel size and compared to the nominal diameter provided by the manufacturer.

We next investigated the dynamic properties of the VACV-GFP-infected cells by using the holographic information, including cell volume. We reasoned that a change in cell volume might result in changes in the density of the cytoplasm, and hence affect the refractive properties of the cell. The accuracy of volumetric measurements based on DHTM was first assessed with polystyrene beads of different nominal sizes ranging from 0.5 to 4 µm (Fig. 4A). Bead size was determined by two different analysis methods, a pixel-based method using the vendor’s software, STEVE, and an object-based 3D-surface segmentation method using Imaris (Fig. 4B). Both procedures yielded similar results, although the object-based method was slightly more accurate.

**FIG 5:**
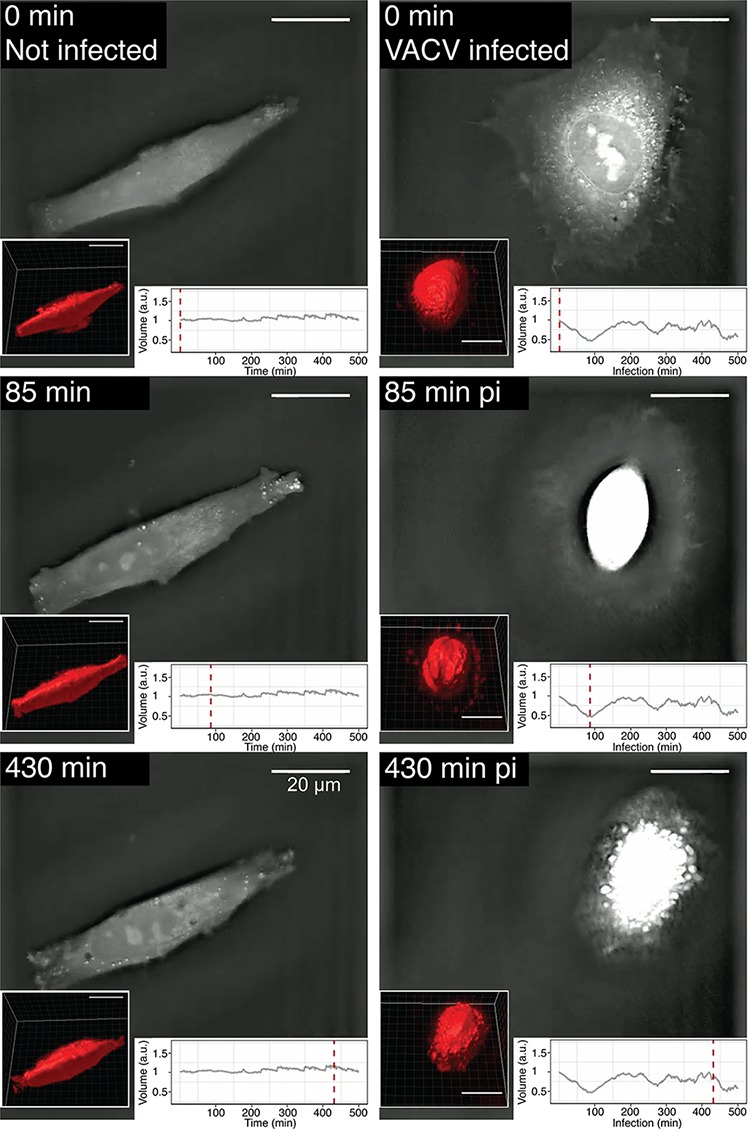
Cell morphology and volume dynamics of VACV-GFP-infected cells, visualized by label-free time-lapse DHTM. HeLa-ATCC cells were infected by VACV-GFP following the cold binding protocol. Cell holograms were acquired every minute for 8 h. RI is shown as gray scale images. Volume measurement was performed using Imaris software by surface fitting and 3D rendering (see the lower left corner of each frame). The plots in the lower right-hand corners show the relative volume (in arbitrary units [a.u.]) normalized to 0 min pi. The red dashed lines correspond to the time points related to the corresponding hologram. Bars, 20 µm. See also Video S2 and Video S3 in the supplemental material.

To measure cell volume by DHTM, we used the image stack segmentation procedure in Imaris. Time-resolved 3D live-cell DHTM imaging of infected and uninfected cells between 0 and 8 h pi indicated that the VACV-GFP-infected cells underwent a series of shrinkage and dilation phases, while the volume of the uninfected cells remained largely constant (Fig. 5, Video S2, and Video S3). The first shrinkage phase peaked at about 100 min pi and reduced the cell volume by about 50%. This contraction was followed by a steady recovery phase restoring the original volume at about 180 min pi. The next shrinkage period started at 230 min and lasted about 40 min, followed by a short recovery phase of about 30 min, and a period of stable volume with minor fluctuations until 440 min pi, when another shrinkage period occurred. We did not detect a correlation between cell shrinkage or expansion with the RIG values, the latter steadily increasing over the course of infection. We conclude that the VACV-GFP infection-induced RIG increase is not caused by generic changes in cell volume.

10.1128/mSphereDirect.00599-18.3 VIDEO S2 Cell morphology and volume dynamics of an uninfected cell visualized using label-free time-lapse holo-tomographic microscopy. HeLa-ATCC cells were treated with cold binding medium, followed by transfer to 37°C. Cell holograms were acquired every minute for 8 h and shown as gray scale images. Volume measurement was performed using Imaris software by surface fitting (3D rendering in the lower left corner of each frame). The volumes relative to the volume at the 0**-**min time point are plotted. The red dashed lines depict the corresponding time points in the volume plot. Scale bar indicates 20 µm. The video is related to Fig. 5. Download Video S2, MOV file, 1.5 MB. Copyright © 2018 Yakimovich et al. 2018 Yakimovich et al. This content is distributed under the terms of the Creative Commons Attribution 4.0 International license.

10.1128/mSphereDirect.00599-18.4 VIDEO S3 Cell morphology and volume dynamics of VACV-infected cell visualized using label-free time-lapse holo-tomographic microscopy. HeLa-ATCC cells were infected with VACV_E/L-GFP virus (MOI of 2) by cold synchronization. Cell holograms were acquired every minute for 8 h and shown as gray scale images. Volume measurement was performed using Imaris software by surface fitting (3D rendering in the lower left corner of each frame), volume relative to 0 min is plotted. The red dashed lines depict the corresponding time points in the volume plot. Scale bar indicates 20 µm. The video is related to Fig. 5. Download Video S3, MOV file, 2.3 MB. Copyright © 2018 Yakimovich et al. 2018 Yakimovich et al. This content is distributed under the terms of the Creative Commons Attribution 4.0 International license.

**FIG 6:**
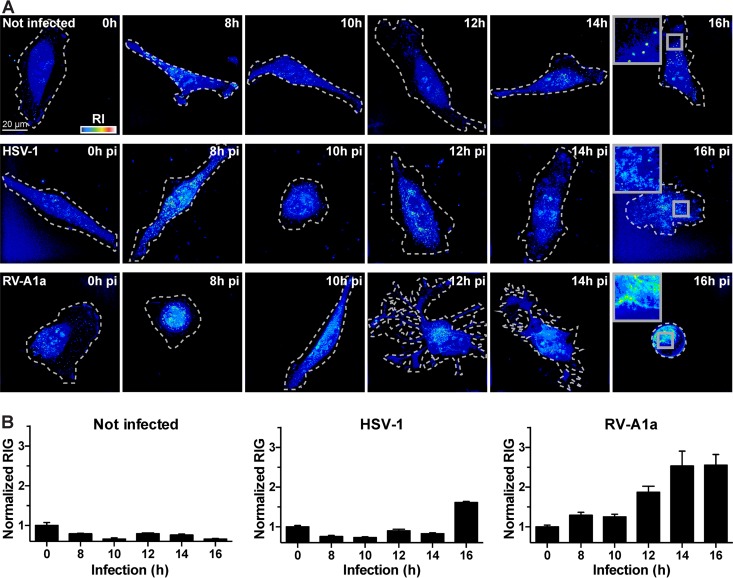
HSV-1 and RV increase cellular RIG late in infection. (A) Representative images of uninfected and infected cells imaged by a DHTM microscope at the indicated time points. HeLa-ATCC cells were infected with HSV-1-GFP (MOI of 10), and HeLa Ohio cells were infected with RV-A1a (MOI of 50). The perimeters of the cells grown on coverslips are outlined with white dashed lines, and the refractive indices are depicted as intensities in a “thermal” lookup table. Images were obtained as holograms, and the 3D stacks are depicted as z-projections of maxima. Bars, 20 µm. (B) Normalized cell RIG of the data shown in panel A. Bars depict mean values ± SEM of at least 10 cells for each condition and time point.

To further explore the correlations of RIG and virus-induced CPE, we recorded RIG in cells infected with HSV-1-GFP harboring a GFP transgene under the constitutively active CMV promoter, and RV-A1a at 0, 8, 10, 12, 14, and 16 h pi (Fig. 6A). Calculations are based on at least 10 cells per time point and condition. In HSV-1-GFP-infected cells, CPE was observed from 8 to 10 h pi on, followed by cell surface roughening at 16 h pi. Remarkably, RIG did not increase despite the onset of CPE prior to 16 h pi (Fig. 6B). Cells infected with RV-A1a showed strong CPE starting from 10 and 12 h pi, involving the condensation of cytoplasm. The RIG of RV-A1a-infected cells steadily increased at 10 to 16 h pi up to about 2.5-fold of the RIG of the uninfected controls. The RIG increase occurred during a time frame when apoptosis, virus-controlled necroptosis, and the loss of cytoskeletal elements, such as F-actin, take place (21–23). Remarkably, RV-A1a-infected cells adopted a transient branched shape at 12 to 14 h pi, before rounding up at 16 h pi (Fig. 6A).

**FIG 7:**
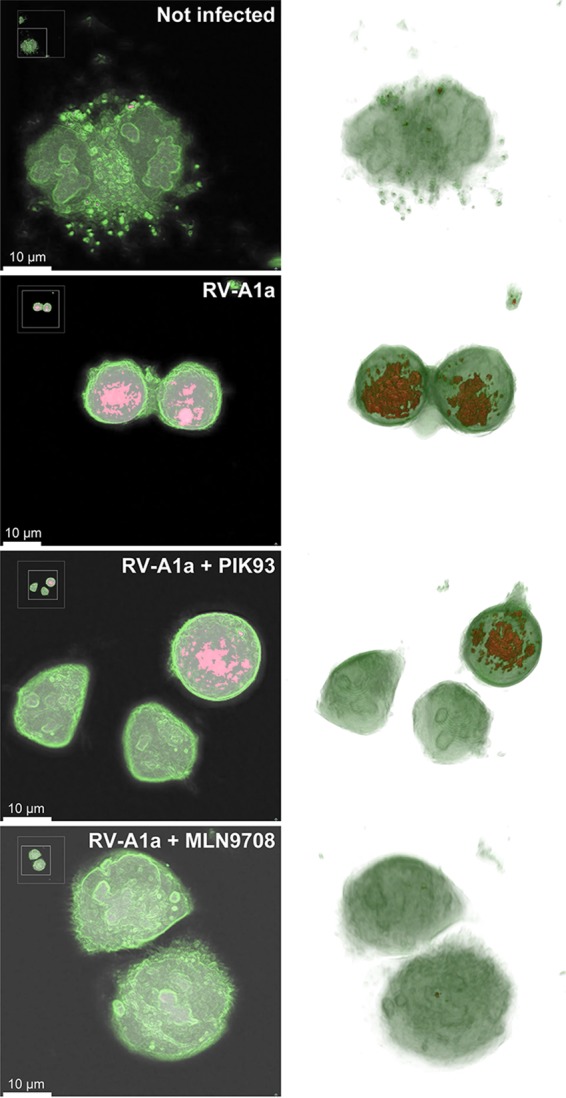
Drug treatment inhibits changes in RIs of RV-A1a-infected cells. Representative images of mock-infected (top panel) or RV-A1a-infected (MOI of 50) HeLa Ohio cells (lower three panels). Cells were treated with either PIK93 (5 µM), or MLN9708 (10 µM), and imaged by DHTM for 8 h at 1-min intervals. Cell membranes are labeled in green, high RI and RIG regions are shown in red. Images in the left column depict central z-slices of reconstructed holograms, and the images in the right column are 3D reconstructions of the holograms. See also Videos S4 to S7. Bars, 10 µm.

**FIG 8:**
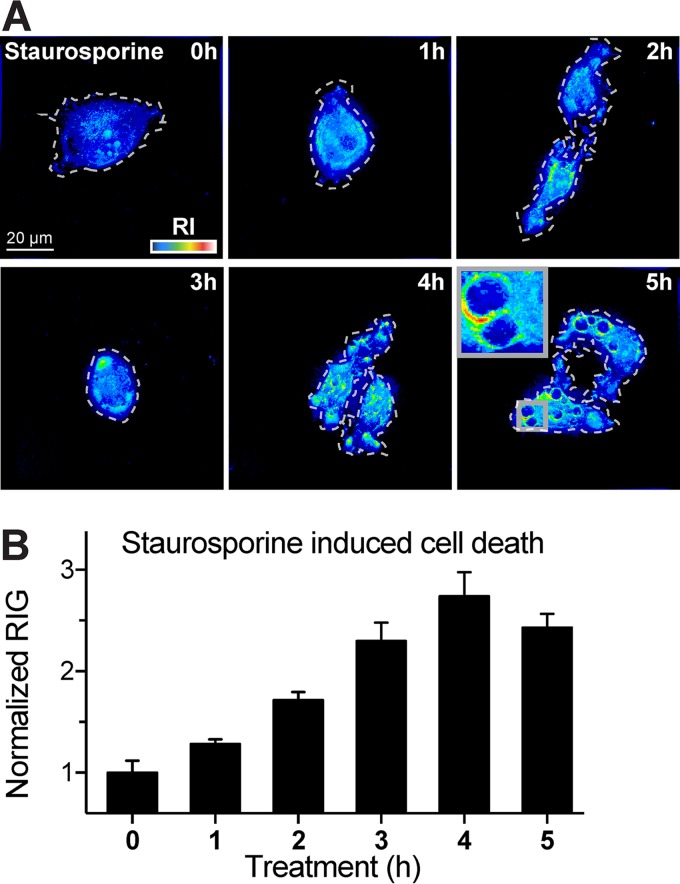
Staurosporine-induced apoptosis rapidly increases the cellular RIG. (A) Representative DHTM images of HeLa-Ohio cells incubated with staurosporine (10 µM) at 37°C for 5 h. Cell perimeters on the glass coverslip are outlined with white dashed lines, and RIs are depicted as intensities in the “thermal” lookup table. Images were obtained as holograms, and the 3D stacks are depicted as z-projections of maxima. Bar, 20 µm. (B) Cell RIG quantification of panel A. RIG values were normalized to 1 at 0 h after the addition. Bars depict mean values ± SEM of at least 10 cells for each condition and time.

Two distinct inhibitors of RV replication were employed to test whether the RIG increase was directly associated with RV-A1a replication. The first inhibitor, PIK-93, blocks phosphatidylinositol-4-kinase class 3beta (PI4K3b) activity, and thereby precludes the lipid remodeling by countercurrent lipid fluxes for virus replication (24). In the absence of PI4K3b activity, viral replication is suppressed, because the altered lipid composition of the replication membrane can no longer support viral host proteins necessary for virus replication (reviewed in reference 25). The second inhibitor, MLN9708, is a second-generation inhibitor of the proteasome, which is required for enterovirus replication (26–28). Both PIK-93 and MLN9807 blocked the appearance of high-RI/RIG structures, although the effect of PIK-93 was incomplete (Fig. 7). The partial effect of PIK-93 on RIG was, however, consistent with the partial reduction of PI4P levels and the incomplete inhibition of RV-A1a replication (24, 29). These results support the notion that an increase in RIG in virus-infected host cells occurs in a virus type-specific manner and that RIG changes are a reliable indicator of early and late CPE. The latter is often associated with virus-induced apoptosis, necrosis, or necroptosis. We therefore tested whether the apoptosis-inducing agent staurosporine (30) also induced a RIG response. DHTM tomography of cells treated with 10 µM staurosporine (Fig. 8A) confirmed that staurosporine induces an apoptotic phenotype as early as 1 h after treatment. Image quantifications indicated that RIG increased up to 4 h after application and slightly decreased at 5 h (Fig. 8B). The extent of the RIG increase reached levels similar to the levels reached upon RV-A1a infection, thereby confirming that apoptotic phenotypes can be tracked by DHTM.

10.1128/mSphereDirect.00599-18.5 VIDEO S4 Drug treatment inhibits RV-A1a-induced RI changes. HeLa-Ohio cells were left uninfected. Holo-tomographic images were acquired at 1-min intervals for 8 h. Cell membranes are labeled in green; high refractive index and refractive index gradient regions are labeled in red. Scale bars indicate 10 µm. Still frames of the video are provided in Fig. 7. Download Video S4, MOV file, 9.0 MB. Copyright © 2018 Yakimovich et al. 2018 Yakimovich et al. This content is distributed under the terms of the Creative Commons Attribution 4.0 International license.

10.1128/mSphereDirect.00599-18.6 VIDEO S5 Drug treatment inhibits RV-A1a-induced RI changes. HeLa-Ohio cells were infected with RV-A1a (MOI of 50) and left untreated. Cell membranes are labeled in green; high refractive index and refractive index gradient regions are labeled in red. Scale bars indicate 10 µm. Still frames of the video are provided in Fig. 7. Download Video S5, MOV file, 5.1 MB. Copyright © 2018 Yakimovich et al. 2018 Yakimovich et al. This content is distributed under the terms of the Creative Commons Attribution 4.0 International license.

10.1128/mSphereDirect.00599-18.7 VIDEO S6 Drug treatment inhibits RV-A1a-induced RI changes. HeLa-Ohio cells were infected with RV-A1a (MOI of 50) and treated with 10 µM MLN9708. Holo-tomographic images were acquired at 1-min intervals for 8 h. Cell membranes are labeled in green; high refractive index and refractive index gradient regions are labeled in red. Scale bars indicate 10 µm. Still frames of the video are provided in Fig. 7. Download Video S6, MOV file, 8.3 MB. Copyright © 2018 Yakimovich et al. 2018 Yakimovich et al. This content is distributed under the terms of the Creative Commons Attribution 4.0 International license.

10.1128/mSphereDirect.00599-18.8 VIDEO S7 Drug treatment inhibits RV-A1a-induced RI changes. HeLa-Ohio cells infected with RV-A1a (MOI of 50) and treated with 5 µM PIK93. Holo-tomographic images were acquired at 1-min intervals for 8 h. Cell membranes are labeled in green; high refractive index and refractive index gradient regions are labeled in red. Scale bars indicate 10 µm. Still frames of the video are provided in Fig. 7. Download Video S7, MOV file, 7.9 MB. Copyright © 2018 Yakimovich et al. 2018 Yakimovich et al. This content is distributed under the terms of the Creative Commons Attribution 4.0 International license.

## DISCUSSION

DHTM is a noninvasive label-free light microscopy technology. It measures the RI of a transparent object by an interference procedure (31–36). DHTM is superior to classical optical techniques, such as tomographic phase microscopy and diffraction phase microscopy (37), because of its high sensitivity, accuracy, and noninvasiveness. The system produces quantitative 3D information at high spatial and temporal resolution. It readily measures the volume of cells and subcellular structures. Furthermore, it can be used to acquire two new dimensions of cell data, RI and RIG, which cannot be readily assessed by other imaging modalities. The methodology is robust and overcomes a series of technical limitations in live-cell pathogen imaging, including the labeling of cellular and viral entities by chemical or genetically encoded fluorophores, and toxicity ensuing imaging (for recent reviews, see references 38 and 39). The noninvasiveness and low laser power required for DHTM underscore the suitability of DHTM for long-term live imaging (40).

Here, we introduce DHTM to virus research by employing three different viruses, VACV of the *Poxviridae*, HSV-1 of the *Herpesviridae*, and RV from the *Picornaviridae*. VACV is an enveloped, double-stranded DNA virus, replicating and assembling particles in the cytoplasm (41). It is used to immunize and protect humans against smallpox caused by variola virus, one of the deadliest viruses to humans (42, 43). VACV exploits apoptotic mimicry to enter host cells through cellular blebbing, that is, protrusions of the cell membrane, implicated in cytokinesis and cell motility (20, 44, 45). VACV-induced morphological changes of host cells include cell rounding, blebbing, and bud-like swellings (18). Picornaviruses are small, positive-sense single-stranded RNA viruses with a nonenveloped icosahedral capsid of 28 to 30 nm in diameter (46). RVs replicate in the cytoplasm on Golgi-derived membranes that are closely associated with the ER, and elicit a strong CPE in tissue culture (23–25). HSV-1 is an enveloped, double-stranded DNA virus, well adapted to human hosts and controlled by innate immunity, including the interferon-induced human myxovirus resistance protein B (MxB) (47). If HSV-1 breaks through the innate host defense, it replicates in the cell nucleus and sheds progeny prior to cell lysis (48, 49). In its latent state, HSV-1 evades the immune system, avoids CPE, and downregulates apoptosis (50, 51). Upon reactivation, HSV-1 is transmitted to the epidermal tissue where it causes lytic infection manifested as epidermal blisters.

We initially used DHTM to determine the volume of cells and became aware of periodic shrinkage and expansion of VACV-infected cells, as well as membrane blebbing at late stages of VACV infections, but not in RV-A1a infections. The cyclic volume changes observed by DHTM in VACV infections may be related to cellular contractility changes observed previously in VACV-infected cells, including cell rounding early and cell flattening late in infection (52). In pathology, cell volume dysregulation contributes to disorders, such as liver insufficiency, diabetic ketoacidosis, hypercatabolism, fibrosing disease, and sickle cell anemia, and regulation of cell volume affects cell proliferation and apoptosis (53). The VACV infection-induced cell volume oscillation implies that VACV regulates membrane trafficking, such as endocytosis and secretion, and possibly also ion channels. For example, the release of potassium, chloride, and bicarbonate ions is known to trigger cell shrinkage, or the accumulation of sodium, potassium, and chloride ions causes cell swelling through the activation of cotransporters, exchangers, or channel proteins (53). In addition, shrinkage and swelling processes involve organic osmolytes, such as sorbitol and glycerophosphorylcholine, which accumulate in shrinking cells and are released in swelling cells. How exactly VACV controls these processes remains an open question.

In addition to the accurate, noninvasive readout of cell volume, the RIGs measured by DHTM have served as an indicator for the granularity of cell structures (54). We show that specific RIG signatures indicate virus-induced CPEs. CPE follows cell shrinkage and blebbing and terminates in cell death (12, 55). In fact, viruses control cell death processes, including apoptosis or necroptosis, for example by diverting upstream regulating kinases, such as RIPK1 in RV-infected cells (1, 23). Other viruses prevent infection-induced apoptosis at early stages, gaining crucial time for production of virus progeny (50, 56–58). At late infection stages, viruses gain virulence by enhancing progeny virus release from the cells (56, 59, 60). This leads to the notion that lytic infection resembles a necrosis-like program, leading to the release of both cellular contents and virus particles (7, 61).

CPEs are elicited not only by a wide range of pathogens, including viruses and bacteria, but they also emerge in malignant cells during transformation and in immune defense by cytotoxic cells for instance (6). Despite such insights, the CPE has remained one of the least understood processes in infection biology and pathology. This is in part due to the fact that a plethora of virus-induced cell reactions tune the infection, including proviral and antiviral signaling and innate immunity response. CPE arises as a result of the many altered processes in an infected cell. It is triggered by a few viral genes introduced into a naive cell and comprises proviral and antiviral components. Our results show that all viruses tested, VACV, HSV-1, and RV-A1a, induced RIG increase over the course of infection, with virus-specific kinetics, extent, and signature. Importantly, the RIG measurements scored CPE independent of cell contractions and blebbing, which cannot be uncoupled in phase-contrast microscopy. This indicates that RIG truly measures new dimensions in CPE that have not been accessible with conventional microscopy analyses. It underlines the superiority of DHTM over phase-contrast microscopy and allows the attribution of virus-specific processes to CPE. In the case of VACV infection, the RIG increase appeared to require late viral gene expression, since RIG changes were impaired by AraC, which inhibits late genes but not intermediate or early genes. For RV, we infer that at the onset of RIG increase at 8 h pi, not only are cytoplasmic membranes being rearranged and expanded for viral replication but also phase separation processes occur in the perinuclear area, a hallmark of RV-infected cells. In the case of HSV-1, the RIG increase was most dramatic at 16 h pi, with large changes in the nucleus and the cytoplasm, indicative of processes associated with virion production and release from the infected cell.

In conclusion, we show that DHTM is suitable to analyze cell biological processes in virus-infected cells at high spatiotemporal resolution, without the need to introduce cell markers or use high-intensity laser light. DHTM has high spatial resolution with potential superresolution options (35, 62). It enhances insights into CPE, a complex phenotype induced by nearly all pathogens, and increasingly mined by high-throughput screening assays (63). DHTM promotes the dynamic analyses of virus assembly, for example in phase separated zones of the cytoplasm and the nucleus, and provides a deeper understanding of the nature of viruses.

While the current state of DHTM technology has provided novel insights into virus-host interactions, the current technology has some limitations. For example, the correlative fluorescence-RI images provided in Fig. 1 are still images acquired with a prototype system and manual image acquisition. An automated stage would increase the statistical power and enable analyses of cell-to-cell variability, for example in correlation with virus replication kinetics and host factor contributions. Simultaneous video acquisition and RI analyses would further facilitate a broad-scale assessment of volume changes of whole cells or subcellular structures. Software implementations allowing for noninvasive simultaneous imaging of multiple cells could enhance the throughput of the technology. Collectively, this may enable future studies to test scenarios, such as how the expression kinetics of viral genes affect RI and RIG or how apoptosis and necrosis induction correlate with commonly used fluorescence markers.

## MATERIALS AND METHODS

### Cell lines and viruses.

HeLa-ATCC cells from the American Type Culture Collection (ATCC) were maintained in Dulbecco modified Eagle medium (DMEM) (GIBCO-BRL) containing 10% fetal calf serum (FCS), nonessential amino acids (NEAA), and penicillin-streptomycin (GIBCO-BRL) at 37°C and 5% CO_2_. All cell cultures were maintained in a cell bank system and kept at low passage numbers for all experiments.

Vaccinia virus strain International Health Department J (VACV-IHD-J) containing early/late (E/L) GFP transgene was kindly provided by J. Mercer (University College London, United Kingdom) (17, 64). To obtain the purified mature virions (MVs), cytoplasmic lysates were pelleted through a 36% sucrose cushion for 90 min with Optima XPN-100 ultracentrifuge (Beckmann Coulter) SW32Ti rotor at 18,000 rpm. The viral pellet was suspended in 10 mM Tris (pH 9.0), and virus was separated from contaminating material on a 25% to 40% sucrose gradient at 14,000 rpm for 45 min. Following centrifugation, the viral band was collected by aspiration and concentrated by pelleting at 14,000 rpm for 45 min. MVs were suspended in 1 mM Tris-HCl (pH 9.0), and the titers of virus were determined (titers in plaque-forming units [PFU] per milliliter) as previously described (65).

RVs were grown in HeLa cells as described previously (66, 67). Cells were inoculated with a lysate from infected cells at 33.5°C overnight. When CPE was visible in 80 to 90% of the cells, the medium was removed, and cells were harvested by scraping and pelleting. Cells were lysed by three freeze/thaw cycles in liquid nitrogen, followed by the addition of 1% NP-40 and homogenization with a Dounce homogenizer. The suspension was centrifuged at 2,500 × *g* for 10 min, and the supernatant was transferred into a new tube. Free RNA was digested by the addition of 150 μg RNase per 10 ml and incubation at 37°C for 30 min. Virus was purified on a CsCl gradient and extensively dialyzed against 140 mM NaCl, 25 mM HEPES, and 5 mM MgCl_2_.

The HSV-1 recombinant strain C12 expressing GFP from the major CMV promoter was kindly provided by S. Efstathiou (University of Cambridge, Cambridge, UK), and used as described previously (47, 68). All virus stocks were stored at −80°C. Infectivity of virus stocks was quantified by high-throughput fluorescence microscopy to determine the amount of inoculum resulting in >95% infected cells. From this, we calculated the MOI based on successful spreading events in plaque titration or TCID_50_ experiments. VACV was titered on HeLa-ATCC cells, RV on HeLa-OHIO, and HSV-1 on A549-ATCC cells, according to earlier protocols (67). Virus-specific MOIs were calculated with TCID_50_s by the Reed-Muench method (69). Note that VACV has an almost 1:1 ratio between early gene expression and spreading infections, while RV has significantly lower frequencies of virus spreading.

### Cold-synchronized infections.

Virus inocula were diluted into HEPES-buffered RPMI (Sigma) containing 10% FCS, NEAA, and penicillin-streptomycin (GIBCO-BRL), added to HeLa cells, and incubated on ice for 30 min. Enough virus was used to infect >95% cells in each of the DHTM experiments. Unbound virus was washed off three times with cold phosphate-buffered saline (PBS) and overlaid with either 37°C warm carbonate-buffered DMEM (GIBCO-BRL) containing 10% FCS, NEAA, and penicillin-streptomycin, or warm HEPES-buffered RPMI (Sigma) containing 10% FCS, nonessential amino acids, and penicillin-streptomycin for automated microscopy or tomographic holography, respectively.

### Automated time-lapse, multisite, multichannel microscopy.

Time-lapse, multisite, multichannel microscopy on HeLa cells grown in 96-well imaging plates (Greiner Bio-One) was performed with an ImageXpress Micro wide-field high-content analysis system (IXM-XL; Molecular Devices) microscope with a synthetic air-to-CO_2_ mixture of 95% to 5%, respectively, in a humidified environment at 37°C with a 20× objective.

### Live label-free holographic tomography.

Live holographic tomography was performed using a 3D Cell Explorer microscope (Nanolive SA, Ecublens, Switzerland). Cells were grown and imaged using 35-mm Ibidi glass bottom µ-Dish dishes (Ibidi GmbH, Germany). During imaging, temperature (37°C) and humidity were controlled using an Ibidi Heating & Incubation System (Ibidi GmbH, Germany), while the pH was maintained by using RPMI containing 20 mM HEPES buffer.

### Cell tracking and volume measurement.

To measure the cell volume, 3D stacks obtained by DHTM were digitally stained and voxel segmented using STEVE (Nanolive SA, Ecublens, Switzerland), exported as TIF files, and imported into Imaris (Bitplane AG, Switzerland). Next, a surface was fitted to the imported 3D voxels aimed at complete volume segmentation. The fitted surface was tracked and enclosed features (volume, centroid position, and centroid speed) were measured with Imaris over the entire duration of the time-lapse experiment. Finally, the volume of each cell in a time-lapse series was normalized to the cell volume at time zero.

To benchmark the volume measurements from holographic tomography, we used polystyrene beads with diameters of 0.5, 0.75, and 1 µm (Fluoresbrite; Polysciences) and 4 µm (Tetraspeck; ThermoFisher). The beads were diluted in PBS, allowed to sediment to the bottom of the dish, and imaged. We next quantified their volume either by voxel segmentation and counting in STEVE or by surface fitting in Imaris (Fig. 4).

### Cell refractive index gradient measurement.

The RI of a material is defined as speed of light in vacuum divided by the speed of light in the particular material. RIG is a computed value describing RI change within a neighborhood of a particular voxel according to the following equation:

(1) $$\text{RIG}=\sqrt{{\left(\frac{{\text{RI}}_{x+1,y,z}-{\text{RI}}_{x-1,y,z}}{2p_{x}}\right)}^{2}+{\left(\frac{{\text{RI}}_{x,y+1,z}-RI_{x,y-1,z}}{2p_{y}}\right)}^{2}+{\left(\frac{{\text{RI}}_{x,y,z+1}-{\text{RI}}_{x,y,z-1}}{2p_{z}}\right)}^{2}}$$

where 2_*p*_*x*__, 2_*p*_*y*__, and 2_*p*_*z*__ are the resolution of the image, and *RI*_*x*±1,*y*±1,*z*±1_ is the RI value of the neighboring pixels.

Measurement of the relative RIG of the cells was performed by “digital staining” in STEVE according to the user manual. The staining was aimed at segmenting at least 95% of the manually determined cellular signal; next, the mean RIG of the digital staining was calculated. Boundaries were adjusted by shifting the RI outside of the 95% constraint. Finally, RIG of each cell in a time-lapse experiment was normalized to the RIG of the cell at the start of the time-lapse experiment.

### Data availability.

Scripts, data, and further materials can be downloaded from the GitHub repository at https://github.com/ayakimovich/DHMViruses.

10.1128/mSphereDirect.00599-18.1 FIG S1 (A) Three representative images of HeLa-ATCC cells inoculated with RPMI at 37°C for 8 h or infected with VACV-GFP at an MOI of 2, followed by DHTM imaging. Images were obtained as holograms and are depicted as projections of maxima along the *z* axes of the 3D stacks. RIs are shown as intensity values in the “thermal” lookup tables. Scale bar indicates 20 µm. (B) Three representative images of uninfected HeLa-ATCC cells, HeLa-ATCC cells infected with HSV-1-GFP (MOI of 10), and HeLa-Ohio cells infected with RV-A1a (MOI of 50), imaged by DHTM at 16 h postinoculation. Images were obtained as holograms, and the 3D stacks are depicted as z-projections of maxima. The refractive indices are depicted as intensities in the **“**thermal” lookup tables. Scale bars indicate 20 µm. Download FIG S1, TIF file, 2.1 MB. Copyright © 2018 Yakimovich et al. 2018 Yakimovich et al. This content is distributed under the terms of the Creative Commons Attribution 4.0 International license.
