# Supplementary figures and images for: Label-Free Digital Holo-tomographic Microscopy Reveals Virus-Induced Cytopathic Effects in Live Cells
Source: mSphere. 2018 Nov 21;3(6):e00599-18. doi: 10.1128/mSphereDirect.00599-18 (PMC6249643; doi:10.1128/mSphereDirect.00599-18)

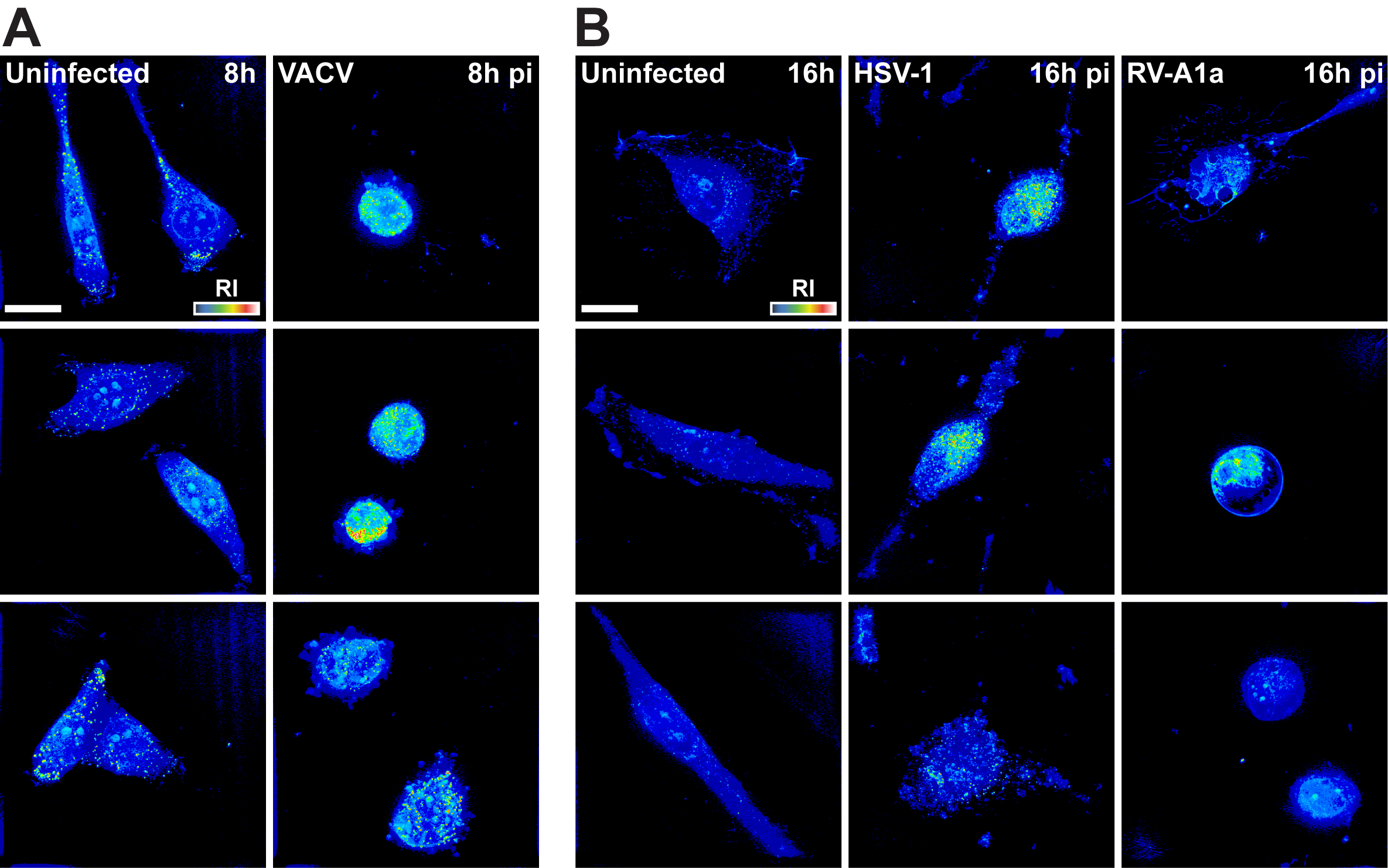

Supplement: FIG S1 [file sph006182707sf1.tif]
